# *Dioscorea quinqueloba* Ameliorates Oxazolone- and 2,4-Dinitrochlorobenzene-induced Atopic Dermatitis Symptoms in Murine Models

**DOI:** 10.3390/nu9121324

**Published:** 2017-12-05

**Authors:** Jonghwan Jegal, No-June Park, Sim-Kyu Bong, Hyun Jegal, Su-Nam Kim, Min Hye Yang

**Affiliations:** 1 College of Pharmacy, Pusan National University, Busan 46241, Korea; puhahaha2027@naver.com; 2 Natural Products Research Institute, Korea Institute of Science and Technology, Gangneung 25451, Korea; parknojune1@naver.com (N.-J.P.); 115044@kist.re.kr (S.-K.B.); 116524@kist.re.kr (H.J.)

**Keywords:** *Dioscorea quinqueloba*, atopic dermatitis, transepidermal water loss, skin hydration, interleukin 4, immunoglobulin E

## Abstract

*Dioscorea quinqueloba* has been used for food substances, as well as in herbal medicines for allergic diseases such as asthma. This study aimed to investigate the anti-atopic dermatitis (AD) effects of the total extract of *D. quinqueloba* rhizomes and active fractionson murine oxazolone- and 2,4-dinitrochlorobenzene-induced models of AD. Specific AD symptoms, such as erythema, ear swelling, and epidermis thickening, were significantly reduced in the oxazolone-mediated AD BALB/c mice upon topical application of *D. quinqueloba* rhizomes 95% EtOH extract (DQ). DQEA (*D. quinqueloba* rhizomes EtOAc fraction) was beneficial for protecting the skin barrier against AD in DNCB-sensitized SKH-1 hairless mice. Decreased total serum IgE and IL-4 levels could be observed in atopic dorsal skin samples of the DQEA-treated group. On the basis of the phytochemical analysis, DQEA was found to contain dioscin and gracillin as its main compounds. Therapeutic applications with *D. quinqueloba* might be useful in the treatment of AD and related inflammatory skin diseases.

## 1. Introduction

Atopic dermatitis (AD) is a pruritic chronic inflammatory skin disease [1]. Worldwide, AD occurs with a prevalence of 2–10% in adults, and up to 15–30% in children [2,3]. Atopic disease is triggered by a variety of allergic factors, including irritants, food, and stress factors [4]. The symptoms of AD include atopic eczema, itching, and dryskin [5]. Intense pruritus is the most common dermatologic feature of AD, thus negatively impacting the health-related quality of life in patients suffering from AD [5,6].

Atopic dermatitis can be categorized into two types, including an extrinsic type (environmental or allergic AD) and an intrinsic type (genetic or non-allergic AD) [7]. Extrinsic AD is the classical type of AD, while the incidence of intrinsic AD is approximately 20% of patients [7,8]. Extrinsic or environmental triggers enhance IgE-mediated sensitization and allergic reaction, thus further contributing to severe forms of skin inflammation in AD [9]. Diverse inflammatory cytokines orchestrate atopic skin inflammation, and the concomitant activation of IL-4 is thought to play a critical role in the pathogenesis of AD [10]. An impaired epidermal skin barrier is also a characteristic feature and causative factor of AD [10]. Skin barrier damage contributes to the high serum IgE level, reduced skin surface hydration, and enhanced transepidermal water loss in patients with extrinsic AD [8,11,12]. Therefore, improvements in skin barrier function with natural moisturizing agents show great potential as a pharmacological target in atopic diseases.

*Dioscorea quinqueloba* Thunb. belongs to the Dioscoreaceae family, and has been cultivated in China, Japan, and Korea as a food. The rhizomes of *D. quinqueloba* have been used in traditional Korean medicine as an alternative therapy for cardiovascular disease, as well as various medical conditions, mainly for arthrosclerosis, myocardial infarction, and asthma [12,13,14]. Analysis of the chemical composition of *Dioscorea* species indicates that the main metabolites are steroidal saponins such as diosin and diosgenin, which have convulsive, local anesthetic and antidiuretic effects [15,16]. Recent reports have shown that the genus *Dioscorea* exhibits various biological properties, including anti-inflammatory, antitumor, and anti-adipogenic activities [17,18,19]. However, studies on the *D. quinqueloba* are comparatively few in number, and furthermore, there have not been any attempts to reveal its anti-atopic effect. This study aimed to investigate the potential therapeutic effects of *D. quinqueloba* on oxazolone- and 2,4-dinitrochlorobenzene (DNCB)-induced murine AD models. Histopathological examination and blood serum analysis, including total IL-4 and IgE levels, were performed to observe anti-atopic properties of *D. quinqueloba*.

## 2. Material and Methods

### 2.1. Plant Material and Extraction

Dried rhizomes of *D. quinqueloba* were purchased from JirisanHanbang Food^®^ (Sancheong, Gyeongnam, South Korea) and identified by Eun Ju Jeong of Department of Agronomy and Medicinal Plant Resources, Gyeongnam National University of Science and Technology. A voucher specimen (PNU-0023) has been deposited in the Medicinal Herb Garden, Pusan National University. *Dioscorea quinqueloba* sample (20 kg) was extracted with 95% EtOH and evaporated under reduced pressure to yield *D. quinqueloba* EtOH extract (DQ) (670 g). The DQ was suspended in distilled water (2 L) and partitioned with EtOAc (4 L) and *n*-BuOH (4 L) to yield *D. quinqueloba* EtOAc fraction (DQEA) (150 g) and *n*-BuOH fraction (DQB) (300 g).

### 2.2. Animals

Six-week-old female BALB/c and SKH-1 hairless mice were purchased from the animal facility of Orient Bio Inc. (Seongnam, Republic of Korea) and housed in an air-conditioned animal room at a temperature of 25 ± 5 °C and 55 ± 5% humidity. Mice were given access to a standard laboratory diet and water ad libitum. All animal experiments were conducted in accordance with the Guide for the Care and Use of Laboratory Animals of the National Institutes of Health (NIH publication No. 85-23, revised 1996) and were approved by the Institutional Animal Care and Use Committee of the KIST (Certification No. KIST-2016-011).

### 2.3. Induction of Topical AD-Like Skin Dermatitis in Mice by Oxazolone and Treatment with DQ

4-Ethoxymethylene-2-phenyl-2-oxazolin-5-one (oxazolone) (1%) dissolved in vehicle (propylene glycol:EtOH = 7:3) was used as sensitizer to induce AD in BALB/c mouse ears according to the previously described method [20]. In brief, the ears of BALB/c mice were sensitized with 20 μL of 1% oxazolone on the first day. After the first challenge, 20 μL of 0.1% oxazolone was repeatedly applied to ears for an additional 3 weeks at 2-day intervals. At the same time, the ears of the BALB/c mice were exposed to 20 μL of 1% DQ daily in the oxazolone-DQ-group for 3 weeks, and the application of 1% DQ was separated by 4 h from that of oxazolone. The normal control animals (CON) were treated with distilled water alone. No substances were applied to the skin surface on the last day of the experiment. On the last day, measurements of skin inflammation signs, including ear swelling and erythema, were carried out.

### 2.4. Induction of AD-Like Skin Lesions in Mice by DNCB and Treatment with DQEA

2,4-dinitrochlorobenzenedissolved in acetone was used to induce AD in SKH-1 hairless mice as previously described with small modification [21,22]. Briefly, the dorsal skin of hairless mice was sensitized by painting 100 μL of 1% DNCB daily for 7 days. After the first challenge, the mice were challenged with 100 μL of 0.1% DNCB for an additional 2 weeks at 3-day intervals. The DNCB-DQEA group animals were painted with 100 μL of 1% DQEA for 2 weeks, and the application of DQEA was separated by 4 h from that of DNCB. The normal control animals (CON) were treated with distilled water alone. No substances were applied to the skin surface on the last day of the experiment. On the last day, mice were sacrificed, and dorsal skin and blood samples were collected for further analysis.

### 2.5. Histology

For histologic examination, the ear or dorsal skin from mice were fixed in 10% formalin and processed for paraffin embedding. Tissue sections (2–3 mm) were then stained with hematoxylin and eosin. Histopathological changes were examined by light microscopy (Olympus CX31/BX51, Olympus Optical Co., Tokyo, Japan) and photographed (TE-2000U, Nikon Instruments Inc., Melville, NY, USA).

### 2.6. Measurement of Transepidermal Water Loss, Skin Hydration, and Skin Surface pH

Skin barrier repair was monitored by estimating transepidermal water loss (TEWL), skin hydration, and skin surface pH. Tewameter TM210 device (Courage and Khazaka, Cologne, Germany) and SKIN-O-MAT (Cosmomed, Ruhr, Germany) were used to evaluate the skin surface of the hairless mice according to the manufacturer’s instructions. TEWL, skin hydration, and skin surface pH were measured once per week after the twice-daily application of DQEA or vehicle.

### 2.7. Measurement of Serum IgE and IL-4 Levels

Blood samples were centrifuged at 10,000 rpm for 15 min at 4 °C, and then serum was collected and stored at −80 °C for further investigations. Total IgE and IL-4 concentration in mouse serum were measured via enzyme-linked immunosorbent assay (eBioscience, San Diego, CA, USA) according to the manufacturer’s instructions.

### 2.8. Measurement of IL-4 mRNA Expression in RBL-2H3 Cells

The rat basophilic leukemia cell line, RBL-2H3, was obtained from the American Type Culture Collection (CRL-2256, Bethesda, MD, USA) and grown in minimum essential medium with Eagle’s salt, supplemented with 10% fetal bovine serum (FBS), 2 mM l-glutamine, 100 U/mL penicillin, and 100 μg/mL streptomycin at 37 °C in a humidified incubator with a 5% CO_2_/95% air atmosphere. The RBL-2H3 cells were treated with dimethylsulfoxide (DMSO), DQ, DQEA, and DQB (10 μg/mL) 30 min before the induction of inflammation with phorbol 12-myristate 13-acetate/ionomycin (PI), which induced a state similar to AD. Controls were treated with DMSO without PMA/ionomycin (PI). After 16 h of treatment, cells were harvested to synthesize cDNA, and mRNA of IL-4 was measured with quantitative real-time PCR (qPCR). Total RNA from the treated cells was prepared with RNAiso Reagent (TaKaRa, Shiga, Japan) according to the manufacturer’s protocol and stored at −80°C until use. Accumulated PCR products were detected directly by monitoring the increase in the reporter dye (SYBR). The expression levels of cytokines in the exposed cells were compared to the expression levels in control cells at each collection time point using the comparative cycle threshold (Ct) method. The sequences of the primers used in this study were: IL-4 forward: 5′-ACC TTG CTG TCA CCC TGT TC-3′; IL-4 reverse: 5′-TTG TGA GCG TGG ACTCAT TC-3′; β-actin forward: 5′-TCA TCA CCA TCG GCA ACG-3′, β-actin reverse: 5′-TTC CT GAT GTC CAC GTC GC-3′. The quantity of each transcript was calculated as described in the instrument manual and normalized to the amount of β-actin.

### 2.9. Chromatographic Conditions

The DQEA sample was analyzed using an Agilent 6530 Accurate-Mass Q-TOF LC/MS system (Agilent Technologies, Palo Alto, CA, USA) for phytochemical characterization. A Poroshell 120 EC-C18 column (3.0 × 100 mm, 2.7 μm, Agilent, Palo Alto, CA, USA) was used for analysis at a flow rate of 0.3 mL/min. The mobile phase consisted of acetonitrile (solvent A) and water (solvent B), using a linear gradient elution: 5–95% A (0–20 min); 10% A (20–30 min). All acquisitions were performed under positive ionization mode. Mass spectra were recorded across the range *m/z* = 100–1500 with accurate mass measurement of all mass peaks.

### 2.10. Statistical Analysis

Data are expressed as the mean ± standard deviation (S.D.). The values were expressed as percent changes from the mean value of the control experiment. Statistical analyses were performed by a one-way analysis of variance (ANOVA) using Statistical Package (SPSS, Inc., Chicago, IL, USA). *p*-values less than 0.05 were considered statistically significant.

## 3. Results

### 3.1. Effects of DQ on AD Symptoms Induced by Oxazolone in BALB/c Mouse Ears

**Figure 1:**
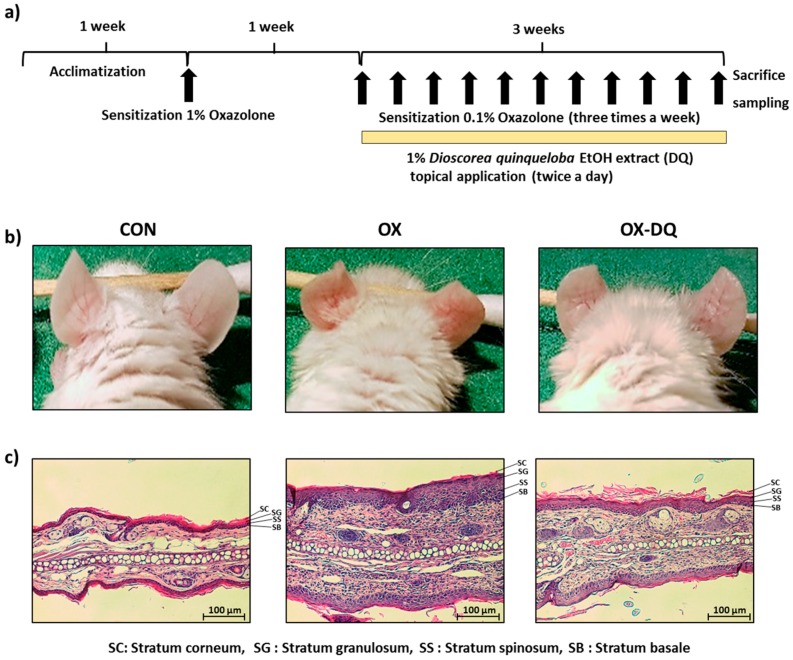
Effects of DQ on the development of oxazolone-induced AD-like symptoms in BALB/c mouse ears and histopathological analysis. CON: control group, OX: oxazolone-treated group, OX-DQ: oxazolone and 1% *D. quinqueloba* EtOH extract-treated group. (**a**) Schematic representation of the experiment; (**b**) Clinical features of AD-like skin lesions; (**c**) Histopathological features of skin lesions. Tissues were excised, fixed in 10% formaldehyde, embedded in paraffin, and sectioned. The sections were stained with hematoxylin and eosin (H&E) (magnification, 100× *g*).

Pathological reactions of AD such as increased ear thickness, erythema, and dryness were observed in the ears of oxazolone-challenged BALB/c mice (Figure 1a). According to the phenotypic observation, ear swelling and erythematic intensity caused by oxazolone were significantly reduced when ears were exposed to 1% DQ for 21 days (Figure 1b). Histologic evaluation also showed that the DQ was efficient at improving AD-like skin lesions in the oxazolone-sensitized mouse ears. Skin thickening and the number of infiltrating lymphocytes were significantly increased in AD mice compared with those in normal mice, and these changes were attenuated by DQ treatment (Figure 1c).

### 3.2. Effects of DQ on Ear Thickness and Serum IgE and IL-4 Levels in Oxazolone-Induced AD BALB/c Mouse Ears

**Figure 2:**
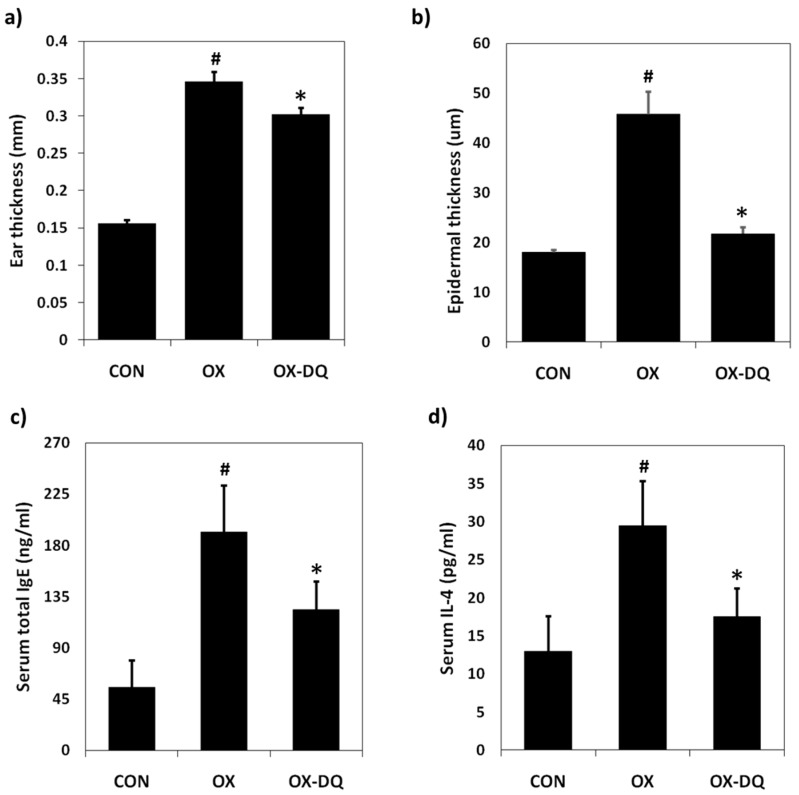
Effects of DQ on the ear thickness and serum IgE and IL-4 levels in oxazolone-induced AD-like symptoms in BALB/c mouse ears. CON: control group, DNCB: DNCB-treated group, DNCB-DQ: oxazolone and 1% *D. quinqueloba* EtOH extract-treated group. (**a**) Ear thickness; (**b**) epidermal thickness; (**c**) Serum total IgE levels; (**d**) Serum total IL-4 levels (D). Results are expressed as the mean ± SEM (standard error of the mean) (*n* = 7). The means ± SEM of two independent experiments are shown. ^#^ *p* < 0.05 vs. control; * *p* < 0.05 vs. oxazolone-treated group.

Increased ear thickness (2.3-fold) and epidermis thickness (2.5-fold) were observed after application of 1% oxazolone to the ears of mice, but these were reversed by treatment with DQ. The ear and epidermal thickness were reduced by 25% and 79%, respectively, on day 28 compared with the oxazolone-treated group (Figure 2a,b). Serum levels of IL-4 and Ig-E were markedly lower in mice of OX-DQ group as compared to the OX group. The 1% DQ application noticeably attenuated total IgE concentration (CON: 52.1 ng/mL, OX: 190.9 ng/mL, and OX-DQ: 118.5 ng/mL) (Figure 2c) and IL-4 concentration (CON: 12.6 pg/mL, OX: 28.6 pg/mL, and OX-DQ: 16.9 pg/mL) (Figure 2d).

### 3.3. Effects of DQEA on AD Symptoms Induced by DNCB in Hairless Mice

**Figure 3:**
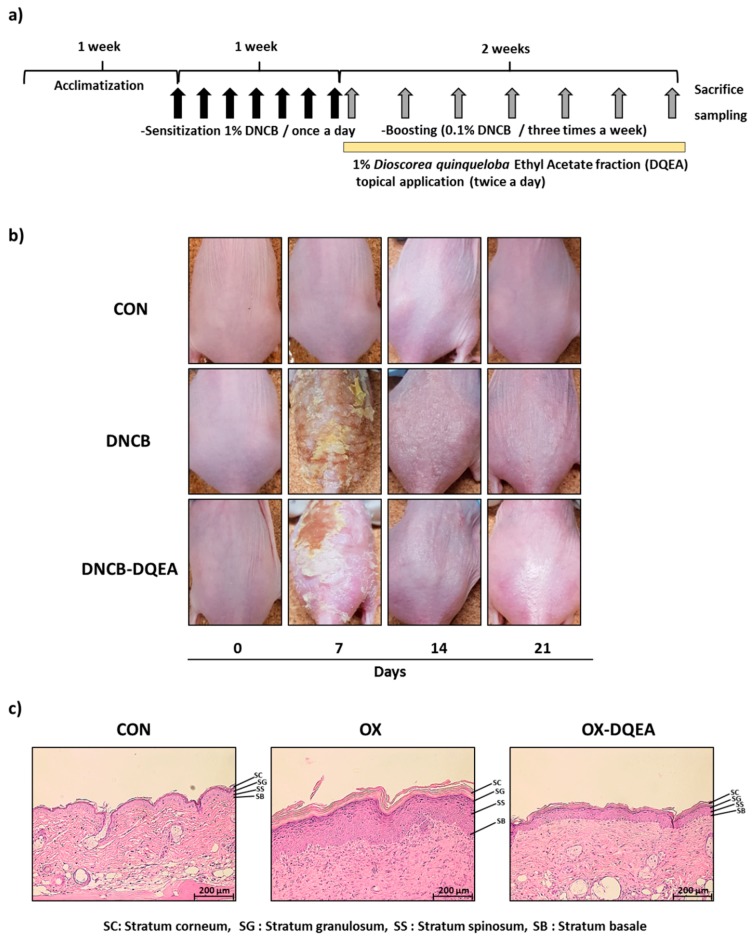
Effects of DQEA on the development of DNCB-induced AD-like symptoms in hairless mice and histopathological analysis. CON: control group, DNCB: DNCB-treated group, DNCB-DQEA: DNCB and 1% *D. quinqueloba* EtOAc fraction-treated group. (**a**) Schematic representation of the experiment; (**b**) Clinical features of AD-like skin lesions; (**c**) Histopathological features of skin lesions. Tissues were excised, fixed in 10% formaldehyde, embedded in paraffin, and sectioned. The sections were stained with hematoxylin and eosin (H&E) (magnification, 100× *g*).

Out of two fractions from DQ (DQEA and DQB), the DQEA exhibited a dramatic drop in mRNA expression of IL-4 genes in RBL-2H3 cells stimulated by PMA/ionomycin (PI) A further experiment was performed to investigate anti-atopic properties of DQEA on AD skin lesions induced by DNCB. For DNCB sensitization, mice were given paintings of 1% DNCB for 3 weeks (Figure 3a). Treatment of 1% DQEA for 2 weeks markedly alleviated DNCB-induced atopy-like dermatitis such as definite erythema, papula, and vesiculation in SKH-1 hairless mice (Figure 3b). Histopathological features of the dorsal skin lesions from DQEA-treated AD hairless mice were shown in Figure 3c. Epidermal thickening by cell hyperplasia, slight spongiosis, and lymphocyte infiltration in the dermis were observed in DNCB-treated control mice, but these were reversed by treatment with DQEA.

### 3.4. Effects of DQEA on Ear Thickness and Serum IgE and IL-4 Levels in DNCB-Induced AD Hairless Mice

**Figure 4:**
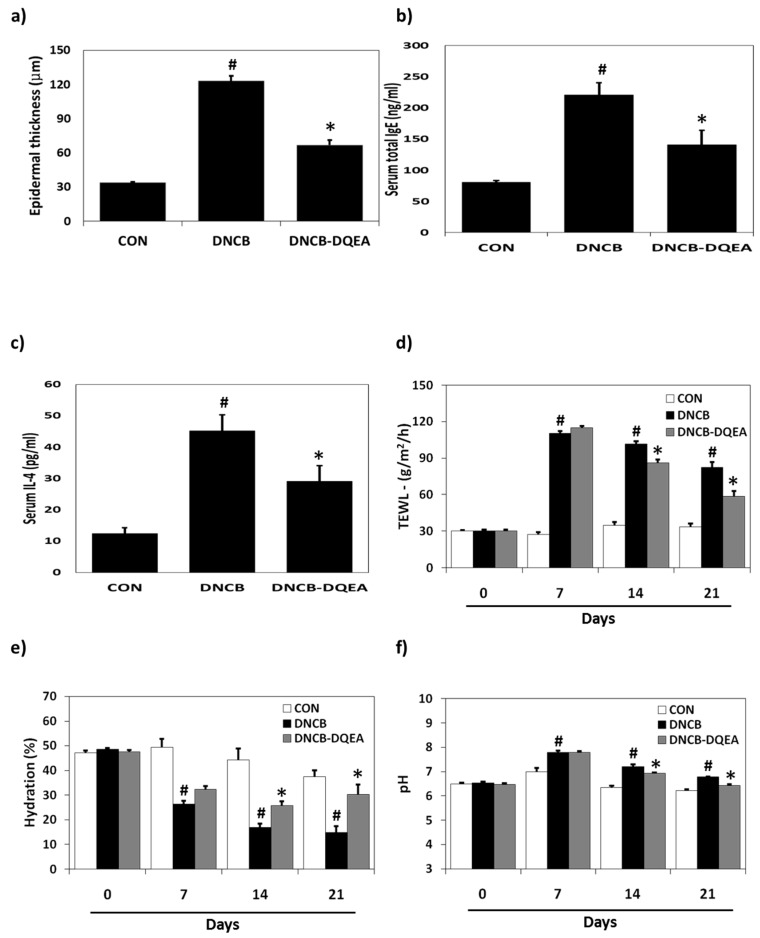
Effects of DQEA on the epidermal thickness, serum IgE and IL-4 levels, and skin barrier function in DNCB-induced AD-like symptoms in hairless mice. CON: control group, DNCB: DNCB-treated group, DNCB-DQEA: DNCB and 1% *D. quinqueloba* EtOAc fraction-treated group. (**a**) Epidermal thickness; (**b**) Serum total IgE levels (**c**) Serum total IL-4 levels; (**d**) TWEL; (**e**) Skin hydration; (**f**) Skin surface pH. Results are expressed as the mean ± SEM (*n* = 7). The means ± SEM of two independent experiments are shown. ^#^ *p* < 0.05 vs. control; * *p* < 0.05 vs. DNCB-treated group.

DNCB-sensitized mice showed a dramatic increase in epidermis thickness, which was significantly reduced in mice treated with DQEA (Figure 4a). DQEA inhibited the DNCB-induced epidermal hyperplasia by 67% in AD hairless mice. The results of serum testing showed that the levels of IgE and IL-4 were increased in the DNCB-stimulated group. The 1% DQEA application noticeably decreased the DNCB-induced serum IgE levels by 65% and IL-4 levels by 57% of DNCB-treated controls (Figure 4b,c).

### 3.5. Effects of DQEA on Skin Barrier Function in DNCB-Induced AD Hairless Mice

Severe skin barrier damage, such as increased TEWL and skin surface PH, and decreased epidermal hydration, was detected in the DNCB-treated control group. This AD-like skin barrier dysfuction was reversed by treatment with 1% DQEA. After 21 days of treatment, DNCB greatly increased TEWL to 79.8 J (g/(m^2^h)), whereas it was markedly reduced to 59.5 J (g/(m^2^h)) by the transdermal application of DQEA (Figure 4d). Consistent with this finding, skin hydration level was decreased in the DNCB-induced group (65.8% decrease) compared with the control group, whereas DQEA increased the hydration level to 68% (Figure 4e). Skin surface pH value was significantly increased in lesional skin, but not in non-lesional skin of AD. Figure 4f revealed that DQEA treatment normalized the altered pH of DNCB-sensitized hairless mouse skin.

### 3.6. The Standardization of DQEA Using the High-Performance Liquid Chromatography/Mass Spectrometry (HPLC/MS)

**Figure 5:**
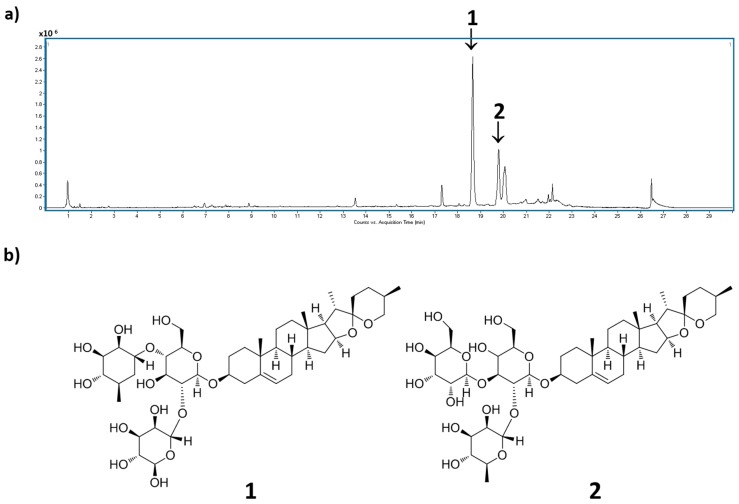
(**a**) HPLC chromatogram of DQEA (**a**) and chemical structures of major components (**b**). Fingerprint analysis of *D. quinqueloba* EtOAc fraction was performed in positive ion mode using HPLC/MS (high-performance liquid chromatography/mass spectrometry). **1**: dioscin, **2**: gracillin.

For the simultaneous determination of the major constituents of DQEA, the optimized chromatographic condition was investigated. The optimal mobile phase, which consisted of acetonitrile/water, was subsequently employed for the analysis of DQEA, and led to a good resolution and satisfactory peak shape. The presence of two compounds, 1: dioscin (*m/z* 868.08 at *t*_R_ 18.66 min) and 2: gracillin (*m/z* 884.08 at *t*_R_ 19.78 min) in DQEA was verified by comparing each retention time and UV spectrum with those of each standard compound and spiking with authentic standards (Figure 5a,b). DQEA was found to contain dioscin (319 mg/g) and gracillin (91.6 mg/g) as major compounds.

## 4. Discussion

Skin barrier dysfunction is one of the primary causes of allergic disorders, and is crucially involved in the pathogenesis of AD [23]. The skin barrier function is mainly disturbed in contact and extrinsic AD, the classical type of AD, which has high prevalence [24]. Impaired permeability barrier function of skin in AD patients enhances penetration of environmental allergens into the skin, thus triggering immunological reactions and inflammation [24,25]. Use of proper moisturizers/emollients to enhance skin hydration is likely to play a key part in management of AD [26]. Natural products are a rich source of medicinal agents, and natural product-related drugs account for over 50% of the most-prescribed drugs in the USA [27]. Substances of natural origin have been widely used for treatment of skin problems due to their therapeutic efficacies in dermatology, which include anti-inflammatory, antimicrobial, and cell-stimulating properties [28]. In addition, plants—including extracts, pure compounds, and phytochemical combinations—are commonly added to moisturizers to improve dry skin conditions in AD patients [28,29]. For this reason, naturally occurring moisturizing agents can be useful in the treatment of at allergic and atopic diseases through improvement skin barrier function [30].

In our preliminary research to find anti-inflammatory materials from plant extracts, the 95% EtOH extract of *D. quinqueloba* (DQ) showed strong IL-4 inhibition in RBL-2H3 cells. The anti-atopic property of DQ was studied using an experimental animal model of AD induced by oxazolone. BALB/c mouse ears were sensitized with oxazolone and treated with DQ subcutaneously for 2 weeks. The results indicated that the topical application of DQ could improve atopic damage in oxazolone-induced BALB/c mouse ears. This finding strongly suggested that the specific fraction from total extract of *D. quinqueloba* is responsible for the anti-atopic effect. For this reason, the DQ was fractionated in order to find the most active principles. Out of two fractions of DQ, the production of IL-4 in RBL-2H3 was only affected by the EtOAc fraction of DQ (DQEA). IL-4 has been considered one of the key proximal cytokines of type 2 inflammation in atopic disease. Impaired barrier function induced by skin sensitizers develops into a pruritic inflammatory skin disease characterized by hyperactivated cytokines of helper T cells [22]. The hyperproduction of IL-4, a key regulatory cytokine for IgE synthesis, is generally detected in various experimental animal models to study AD [31]. In addition, transgenic mice expressing epidermal IL-4 have been reported to develop skin inflammation reproducing all key features of human AD [27,32]. Broad application of DQEA to dorsal skins of DNCB-sensitized SKH-1 hairless mice attenuated severe atopic symptoms such as erythema and lichenification. Taken together, it was concluded that *D. quinqueloba* improves severe AD symptoms in both oxazolone- and DNCB-induced AD mice as a potent IL-4 inhibitor.

Stratum corneum hydration and transepidermal water loss are considered as a marker of the inside-outside skin barrier [33]. The water-holding capacity of the *D. quinqueloba* sample in atopic skin was evaluated by measuring both epidermal hydration and TEWL. Barrier repair was delayed and skin hydration was impaired in DNCB-sensitized mice. Treatment with DQEA significantly increased skin hydration and decreased TWEL and skin surface pH in DNCB-induced AD hairless mice. Atopic skin normally shows a defective skin barrier function, both in rough and in clinically healthy skin [34]. Emollient enhancement of the skin barrier from DQEA-applied AD animals was observed after 3 weeks of treatment. Therefore, DQEA might be an appropriate material for improving barrier function and dry skin as a skin therapeutic agent for AD. An elevated serum level of IgE is a main feature of AD and IL-4 acts as a key cytokine in the process of atopic inflammation [35].DQEA also attenuated IgE hyperproduction and epidermal overexpression of IL-4, resulting in the prevention and treatment of Th2-dominated inflammation in AD-like skin lesions. Based on the results, we propose that DQEA corrects skin barrier dysfunction and early inflammation in murine AD models.

Standardization of *D. quinqueloba* is necessary to provide information on quality standards of natural product-derived drug development. Phytochemical screening, using steroidal saponins as bioactive marker compounds, was performed to standardize the DQEA sample. Steroidal saponins have long attracted scientific attention, due to their structural diversity and significant biological activities [36]. Dioscin and disogenin, steroidal saponins obtained from *Dioscorea* species, are currently being considered as an important starting material for the industrial production of steroid drugs [37]. Phytochemical analysis of the DQEA resulted in the presence of dioscin and gracillin as major components.

## 5. Conclusions

In conclusion, topical treatment with *D. quinqueloba* total extract was protective against oxazolone-induced AD-like lesions in mice ears. Bioassay-guided fractionation of the DQ led to the finding of the most biologically active fraction, DQEA. Clinical symptoms of AD, such as pruritus, erythema, fissuring, and lichenification (skin thickening), were significantly reduced in the DQEA-treated mice. The DQEA improved skin barrier dysfunction and suppressed the overproduction of serum IgE and IL-4 in murine DNCB-sensitized atopic models. Based on the fingerprint analysis, dioscin, and gracillin were confirmed to be the major active constituents of DQEA. Further study is warranted to identify the therapeutic effects of DQEA against AD symptoms in human skin.
